# Impact of miR-155 rs767649 Polymorphism on Rheumatoid Arthritis Activity in Egyptian Patients

**DOI:** 10.7759/cureus.49297

**Published:** 2023-11-23

**Authors:** Mohamed A Elghouneimy, Marwa A Ramadan, Enas A Farrag, Hanan F Ibrahim, Seham K Khirala, Nora Seliem, Sammar A Kasim, Eman M Moazen, Asmaa A Attia, Faten I Mohammed, Aya A Ghamry

**Affiliations:** 1 Plastic Surgery, Nile Insurance Hospital, Cairo, EGY; 2 Clinical Pathology Department, Faculty of Medicine for Girls, Al-Azhar University, Cairo, EGY; 3 Medical Microbiology and Immunology Department, Faculty of Medicine for Girls, Al-Azhar University, Cairo, EGY; 4 Biochemistry Department, Faculty of Medicine for Girls, Al-Azhar University, Cairo, EGY; 5 Internal Medicine Department, Faculty of Medicine for Girls, Al-Azhar University, Cairo, EGY; 6 Department of Chest Disease, Faculty of Medicine, Al-Azhar University, Cairo, EGY; 7 Physiology Department, Faculty of Medicine for Girls, Al-Azhar University, Cairo, EGY

**Keywords:** disease activity, rs767649, mir-155, microrna, rheumatoid arthritis

## Abstract

Background: Rheumatoid arthritis (RA) is a chronic inflammatory condition that impacts not only the musculoskeletal system but also various other systems in the body, including the cutaneous, ocular, respiratory, cardiovascular, and circulatory systems. MicroRNAs (miRNAs) are a class of naturally occurring and highly conserved transcripts that primarily function in the regulation of gene expression. They accomplish this by facilitating the degradation of messenger RNA (mRNA) or by repressing mRNA translation. miRNAs are well-known regulators of a variety of cellular processes. Therefore, we aimed to detect the impact of miR-155 rs767649 polymorphism on RA activity.

Methods: This case-control study included 66 Egyptian patients with RA who visited Al-Zhraa University Hospital, Internal Medicine Department, Cairo, Egypt, and 50 apparently healthy control subjects matched for age and sex. The participants were subjected to full clinical evaluation, including assessments of the disease activity score (DAS), erythrocyte sedimentation rate (ESR), liver and kidney function, anti-cyclic citrullinated peptide antibody (anti-CCP), and miR-155 polymorphism using real-time polymerase chain reaction (PCR).

Results: In the RA group, the majority (98.5%) were female, with a mean age of 43 years, while in the control group, 94% were female, with a mean age of 43.4 years. Comparison of laboratory parameters indicated significantly lower hemoglobin levels, higher ESR, and higher serum creatinine and anti-CCP levels in the RA group than in the control group. The RA group had a significantly higher frequency of TT genotypes and significantly lower frequencies of TA and TT genotypes than the control group. Considering the TT genotype and T allele as references, TA, AA, and TA/AA genotypes in the dominant model; AA in the recessive model; and A allele were significantly associated with protective effects against RA development (p<0.05, odds ratio<1).

Conclusion: rs767649, the functional variant of miR-155, plays an important role in susceptibility to the increased risk of RA, suggesting that miR-155 can be used as a therapeutic target for the treatment of Egyptian patients with RA.

## Introduction

Rheumatoid arthritis (RA) is one of the debilitating autoimmune disorders characterized by chronic inflammation of joints with erosive arthritis [1]. Although the etiology of RA is not clearly known, many factors are implicated in susceptibility to RA, including environmental, genetic, and immunological factors [2]. The clinical diagnosis of RA is based on the revised American College of Rheumatology/European League Against Rheumatism (ACR/EULAR) classification criteria, including evaluation of clinical and serum factors, such as anti-cyclic citrullinated peptide (anti-CCP) antibody and rheumatoid factor (RF) [3]. MicroRNAs (miRNAs) are conserved, small noncoding RNAs with approximately 19-24 nucleotides that play an important role in the regulation of post-translational modifications and in various biological processes, such as homeostasis of the immune system, differentiation of T helper cells, maintenance of tolerance, and development of immune cells [4]. Dysregulated expression of miRNA has been observed in various disease states, such as inflammation, autoimmunity, cardiovascular diseases, and cancer [5]. miRNAs are considered potential targets for therapy in many inflammatory and autoimmune diseases, including RA [6]. MiR-155 is one of the most commonly studied miRNAs and is a critical modulator of immune responses [4]. MiR-155 is also a marker of disease activity in patients with RA [7]. Alterations in miR-155 expression can lead to abnormal functioning of the immune system and, subsequently, the occurrence of autoimmune diseases [4].

Single nucleotide polymorphism (SNP) is a common genetic variation that occurs in a genome [8]. Several studies have shown that SNPs largely affect the stability, targeting activity, and functioning of miRNAs, in turn leading to disease development [9,10]. This study aimed to examine the relationship between miR-155 polymorphism and RA activity.

## Materials and methods

Settings and participants

This case-control study included 65 Egyptian patients with RA, who visited Al-Zhraa University Hospital, Internal Medicine Department, Cairo, Egypt. In addition, 50 apparently healthy control subjects, matched for age and sex, were included. Patients were diagnosed with RA according to the American College of Rheumatology/European League Against Rheumatism (ACR/EULAR) classification [11]. Patients with other autoimmune diseases, such as systemic lupus erythematosus, were excluded. Informed consent was obtained from all the participants. The Research Ethics Committee (REC) of Al-Azhar University, Faculty of Medicine for Girls, approved the protocol of the study (approval number: 2022091520).

All participants were subjected to clinical evaluations (full history taking and general examinations including assessment of disease activity score (DAS)), including complete blood count (CBC), erythrocyte sedimentation rate (ESR), liver and kidney function, anti-cyclic citrullinated peptide antibody (anti-CCP), and miR-155 polymorphism using real-time polymerase chain reaction (PCR).

Blood sampling

In all, 7 mL of venous blood was collected from all participants and subjected to the following processes: 3 mL was collected in a serum vacutainer tube left for 10 minutes and centrifuged at 3000 rpm for five minutes. The separated serum was used for the estimation of liver and kidney functions and anti-CCP. Next, 2 mL was collected in an ethylenediaminetetraacetic acid (EDTA)-containing tube for complete blood count and ESR; 2 mL was collected in an EDTA-containing tube and stored at -20°C until real-time quantitative PCR (q-PCR). CBC was performed using a fully automated hematology analyzer (Sysmex KX21N, Kobe, Japan). Liver and kidney function tests were conducted using a fully automated analyzer (Cobas c311, Germany).

Detection of anti-CCP

The level of anti-CCP antibody was determined using an enzyme-linked immunosorbent assay (ELISA) kit (INOVA Diagnostics QUANTA Lite™ CCP3.1 IgG/IgA ELISA kit (San Diego, CA, USA). The assay utilized a synthetic, CCP antigen that is recognized for its high sensitivity and specificity in the detection of antibodies. The microwells were initially coated with the specific antigen of interest. Following this, the succeeding stages of the experiment required introducing calibrators, controls, and diluted patient samples into the microwells. During the initial incubation period, the samples exhibited binding of anti-CCP antibodies to the antigen. An enzyme-labeled anti-human antibody conjugate was added after thorough washing to eliminate unattached proteins. The conjugate that was bound was observed by employing a chromogenic substrate, and the resultant intensity of color was directly correlated with the concentration of anti-CCP antibodies present in the specimen. The sample was considered negative if <20 U, weak positive if 20-39 U, moderate positive if 40-59 U, and strong positive if ≥60 U.

DNA extraction

DNA was extracted from whole-blood samples using a Gene JET DNA purification Mini kit (Thermo Scientific, Catalog No. 4351379, Lithuania, Vilnius) according to the manufacturer's instructions [12]. This kit yields purified nucleic acids that can be used in any molecular assay. The selective binding properties of the silica-based membrane allow the DNA to bind specifically to it and the contaminants to pass through. Furthermore, PCR inhibitors, such as divalent cations and proteins, can be completely removed in two efficient wash steps, leaving pure DNA to be eluted in a buffer provided with the kit.

Detection of gene polymorphism

DNA was detected by real-time q-PCR using the rs767649 PCR kit with a Rotor-Gene Q cycler (Qiagen, Germany). Genotyping was carried out using a real-time PCR system (Thermo Scientific, Catalog No. 4351379, Vilnius, Lithuania) and the following assay probe sequence: ATATAACATTATCAAAAACACTG (A/T) CACTTTTCTGAGTGCTCTAATCAGG.

The q-PCR technique relies on the identification of fluorescence generated by a reporter molecule, the intensity of which amplifies as the reaction progresses. The fluorescence can be readily detected using a camera throughout each cycle of the PCR process. The emitted signal is then captured by a detector, converted into a digital signal, and subsequently displayed on a computer screen. In addition, the method utilizes a set of primers that are complementary to the target sequence of interest, and these primers are extended by the DNA polymerase [13].

Scatter graph analysis

A scatter graph was plotted to determine the relationship between the variables of interest. This type of analysis enables a quick and simple evaluation of obtained values through a graph. The software can classify samples according to their genotype, but in this study, it was not possible to set the threshold value manually. The allelic discrimination was based on terminal fluorescence acquired in the last cycle of qPCR, and it could be adjusted manually.

Statistical analysis

Our data were analyzed by IBM SPSS Statistics for Windows, version 20 (released 2018; IBM Corp., Armonk, New York, United States). Shapiro-Wilk test was used to test normality. Mean ± standard deviation (SD) and median (range) were used to describe quantitative variables, while numbers and percentages were used to present the categorical variables. The chi-square test was applied to compare qualitative categorical clinical variables between groups. Independent sample t-test and Mann-Whitney U test were used to compare the two groups regarding the quantitative normally distributed and not normally distributed variables, respectively.

Odds ratios (ORs) with 95% confidence intervals (CIs) were calculated. Pearson's correlation test assessed the correlation among the measured parameters. We performed a regression analysis to determine the predictors of RA susceptibility and activity. Statistical significance was set at P<0.05.

## Results

Demographics data and baseline laboratory values

In the RA group, 65 (98.5%) were female and the mean age was 43 (SD, 9.5) years, while in the control group, 47 (94%) were female and the mean age was 43.4 (SD, 6.8) years. There was no significant difference between the two groups in age and sex distribution (P=0.809 and 0.313, respectively) (Table 1).

**Table 1:** Comparison of age and sex distribution between the groups Data are presented as mean (SD) and median (range) or numbers and percentages. p<0.05 is considered significant. SD: standard deviation; RA: rheumatoid arthritis; N: number

		Control N=50		RA N=66		P
Age (years)	Mean ±SD	43.4	6.8	43.0	9.5	0.809
	Range	35	62	25	60	
Male	N (%)	3	6.0%	1	1.5%	0.313
Female	N (%)	47	94.0%	65	98.5%	

The results of the comparative analyses of the CBC parameters, biochemical parameters, and anti-CCP between the groups are presented in Table 2. The RA group showed significantly lower hemoglobin (11.7 vs. 12.2 g/dL; P=0.017) levels and higher ESR (34.7 vs. 14 mm/h; P<0.001), serum creatinine (1.2 vs. 0.9; P<0.001), and anti CCP levels (118.2 vs. 75.4; P<0.001) than the control group. However, there was no significant difference between the two groups in other CBC parameters and alanine transaminase and serum albumin levels.

**Table 2:** Comparison of CBC parameters, biochemical parameters, and anti-CCP levels between the groups Data are presented as mean (SD) and median (range) or numbers and percentages. p<0.05 is considered significant. SD, standard deviation; RA: rheumatoid arthritis; N: number; HB: hemoglobin; WBCs: white blood cells; ALT: alanine transaminase; anti-CCP: anti-cyclic citrullinated peptide; ESR: erythrocyte sedimentation rate

		Control N=50		RA N=66		p
HB (g/dL)	Mean ± SD	12.2	0.8	11.7	1.3	0.017
	Median, range	12.1	11.2-14.5	11.8	6.8-14.9	
WBCs (X10^9^/L)	Mean ± SD	7.5	2.0	7.1	2.5	0.236
	Median, range	7.5	4-11.4	6.9	3.3-16.2	
Neutrophils (X10^9^/L)	Mean ± SD	4.0	1.5	4.1	1.6	0.843
	Median, range	4.0	1.2-7	3.9	1.2-8.8	
Lymphocytes (X10^9^/L)	Mean ± SD	2.2	0.6	2.2	1.1	0.242
	Median, range	2.4	1.1-3.2	2.0	1.2-8.6	
Platelets (X10^9^/L)	Mean ± SD	256.7	93.7	276.5	79.7	0.185
	Median, range	244	128-478	277	119-431	
ALT (U/L)	Mean ± SD	22.6	8.4	27.1	8.3	0.109
	Median, range	24	12-41	28	11-45	
Albumin (g/dL)	Mean ± SD	4.3	0.2	4.2	0.6	0.102
	Median, range	4.3	4-4.7	4.0	3.3-5.3	
Creatinine (mg/dL)	Mean ± SD	0.9	0.1	1.2	0.3	<0.001
	Median, range	0.89	0.71-1.01	1.21	0.61-1.91	
Anti-CCP	Mean ± SD	75.4	15.8	118.2	33.5	<0.001
	Median, range	73	45-98	114	76-321	
ESR (mm/h)	Mean ± SD	14.0	5.0	34.7	14.4	<0.001
	Median, range	15	5-28	32	11-78	

Table 3 summarizes all the clinical data of the RA group. The mean duration of disease in was eight (SD, 3.6) years, with a mean DAS score of 3.9. Most patients (42, 63.6%) had moderate disease activity, and 21.2% had low disease activity. Meanwhile, a few patients showed remission (6.1%), and a few showed high disease activity (9.1%).

**Table 3:** Clinical data of the RA group Data are presented as mean (SD) and median (range) or numbers and percentages. RA: rheumatoid arthritis; SD: standard deviation; DAS: disease activity score

		RA N=66	
Duration of disease	Mean ± SD	8.0	3.6
	Median, range	8	2-19
DAS score	Mean ± SD	3.9	1.1
	Median, range	3.9	1.8-8
Remission	N, %	4	6.1%
Low disease activity	N, %	14	21.2%
Moderate disease activity	N, %	42	63.6%
High disease activity	N, %	6	9.1%

Association of the rs767649 genotype and allelic polymorphisms with other parameters

Frequency of the rs767649 Genotype and Allelic Polymorphisms

The results of the comparative analyses of the frequency of the rs767649 genotype and allelic polymorphisms between the RA and controls groups are presented in Table 4. The frequencies of rs767649 TA and AA genotypes were significantly higher in the control group than in the RA group (P=0.040 and 0.007, respectively). In addition, the TA/AA (dominant) and AA (recessive) genotypes were significantly more frequently observed in the control group than in the RA group (OR=0.492, 95% CI=0.302-0.801, P=0.004 vs. OR=245, 95% CI=0.078-0.764, P=0.015). Similarly, the A allele was more frequently observed in the control group than in the RA group (32% vs. 12.9%; P < 0.001). The TA and AA genotypes, TA/AA (dominant), and AA (recessive) were all protective against RA development.

**Table 4:** Frequencies of the rs767649 genotype and allelic polymorphisms between the case and control groups Data are presented as numbers and percentages. Logistic regression analysis was used. Reference genotype and allele were retrieved from the National Center for Biotechnology Information database. p<0.05 is considered significant. N: number; OR: odds ratio; CI: confidence interval; A: adenine; T: thiamine

rs767649		Control N=50		Cases N=66		P value	OR (95% CI)	
		N	%	N	%			
Genotypes	TT	25	50.0	50	75.8		Reference	
	TA	18	36.0	15	22.7	0.040	0.580	0.345-0.975
	AA	7	14.0	1	1.5	0.007	0.206	0.065-0.651
Dominant	TT	25	50.0	50	75.8		Reference	
	TA/AA	25	50.0	16	24.2	0.004	0.492	0.302-0.801
Recessive	TT/TA	43	86	65	98.5		Reference	
	AA	7	14.0	1	1.5	0.015	0.245	0.078-0.764
Allele	T	68	68.0	115	87.1		Reference	
	A	32	32.0	17	12.9	<0.001	0.486	0.324-0.729

Comparison of rs767649 Genotypes According to Laboratory Parameters

The anti-CCP levels were significantly different between genotypes in the RA group (P<0.001), with higher levels in the TT genotype subgroup than in the TA genotype group; however, no significant difference was observed between the different genotype subgroups in the control group (P=0.442) (Table 5).

**Table 5:** Comparison of serum anti-CCP levels according to rs767649 genotypes between the RA and control groups Data are presented as mean (SD) and median (range) or numbers and percentages. p<0.05 is considered significant. RA: rheumatoid arthritis; SD: standard deviation; anti-CCP: anti-cyclic citrullinated peptide antibody

			Anti-CCP				
		N	Mean	±SD	Median	Range	P value
RA	TT	50	127.3	33.4	117	106-321	<0.001
	TA	15	90.7	9.3	89	78-105	
	AA	1	76	NA	76	NA	
Controls	TT	25	73.5	16.9	68	45-98	0.442
	TA	18	75.6	15	73	47-98	
	AA	7	82	14.4	87	66-98	

In addition, there was a significant difference in the ESR levels between the different genotype subgroups in the RA group, with higher levels in the TT genotype subgroup than in the TA genotype subgroup (P<0.001). However, no significant difference between genotypes was detected in any of the CBC parameters (Table 6).

**Table 6:** Comparison of CBC parameters and ESR according to rs767649 genotypes in the RA group Data are presented as mean (SD) and median (range). p<0.05 is considered significant. SD, standard deviation; RA: rheumatoid arthritis; N: number; HB: hemoglobin; WBCs: white blood cells; ESR: erythrocyte sedimentation rate

		RA cases					
		TT N=50		TA N=15		AA N=1	p
HB (g/dL)	Mean ± SD	11.8	1.3	11.6	1.2	8.5	0.2308
	Median, range	11.9	6.8-14.9	11.7	10-13.3	8.5	
WBCs (x10^9^/L)	Mean ± SD	7.2	2.6	6.7	2.4	7.8	0.745
	Median, range	6.9	3.3-16.2	6.5	3.4-12	7.8	
Neutrophils (x10^9^/L)	Mean ± SD	4.2	1.7	3.7	1.3	5.5	0.412
	Median, range	3.9	1.2-8.8	3.9	1.7-5.5	5.5	
Lymphocytes (x10^9^/L)	Mean ± SD	2.3	1.1	2.2	0.8	1.9	0.905
	Median, range	2.0	1.2-8.6	2.0	1.2-4.3	1.9	
Platelets (x10^9^/L)	Mean ± SD	276.4	79.9	274.9	84.3	307.0	0.869
	Median, range	273	124-431	293	119-404	307.0	
ESR (mm/h)	Mean ± SD	39.7	12.9	19.4	1.8	11	<0.001
	Median, range	35	21-78	20	15-21	11	

Comparison of rs767649 Genotypes According to Clinical Parameters

**Figure 1:**
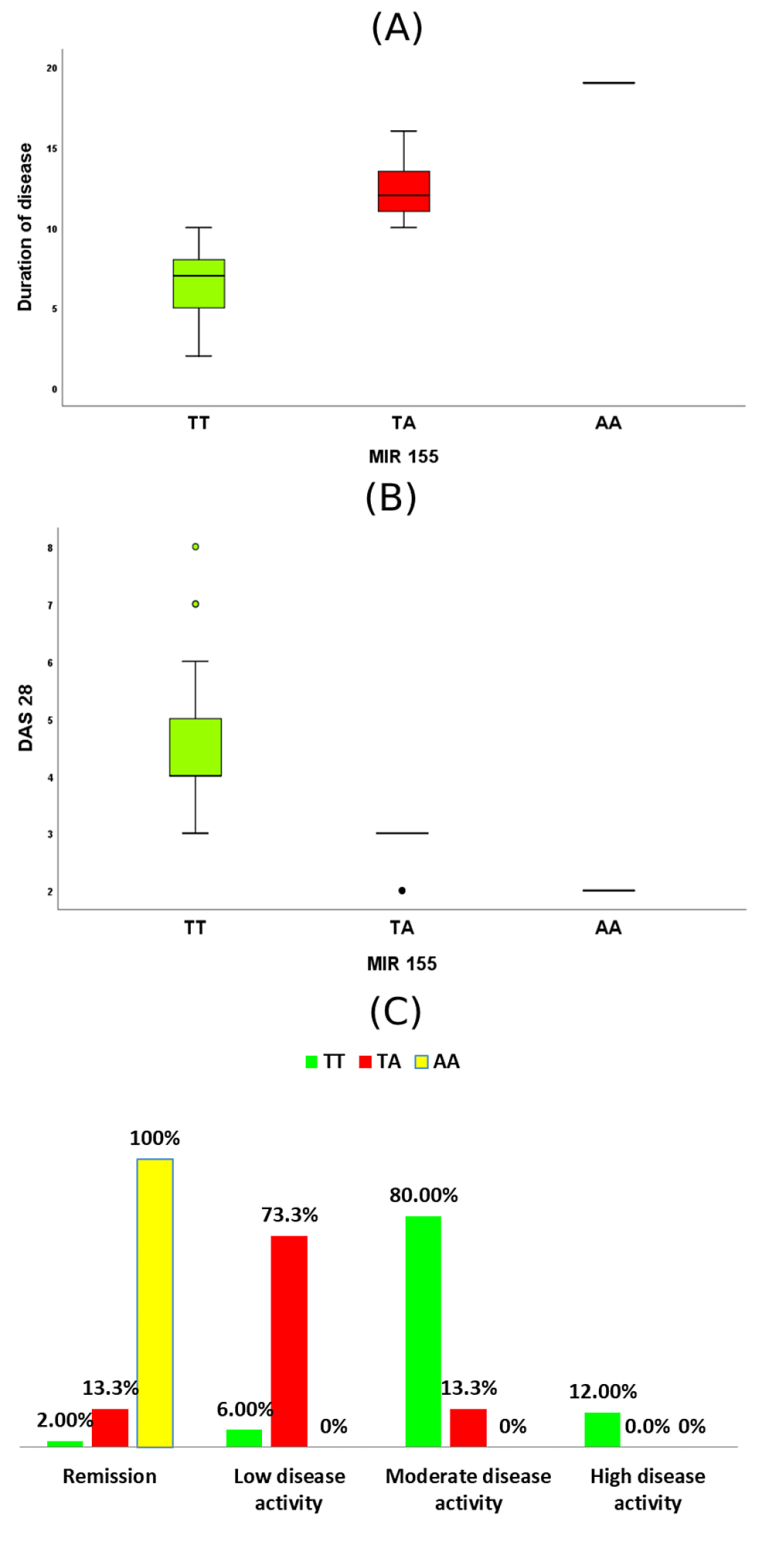
Comparison of different rs767649 genotypes in the RA group: A, disease duration; B, DAS 28; C, disease activity/grade RA: rheumatoid arthritis; DAS: disease activity score

There was a statistically significant difference between the three rs767649 genotypes and all clinical parameters. As shown in Figure 1, the disease duration was significantly higher in the AA genotype subgroup (19 years) than in the TA genotype subgroup (mean (SD): 12.4 (1.9) years; P<0.001). Furthermore, the DAS was significantly higher in the TT genotype subgroup (mean (SD): 4.4 (1)) than in the TA genotype subgroup (mean (SD): 2.9 (0.4); P<0.001). In addition, the patient who had the AA genotype was readmitted, so the readmission rate in this subgroup was 100% compared to 13.3% in the TA and 2.0% in the TT genotype subgroups. Most of the patients with the TA genotype had lower disease activity (73.3%), and most of the patients with the TT genotype had moderate disease activity (80.0%). The difference in disease activity among the three genotypes was statistically significant (P<0.001).

Prediction of outcomes

Table 7 presents the results of regression analysis of the predictors of RA susceptibility. Univariable analysis revealed significant associations of RA susceptibility with the ESR, anti-CCP, and rs767649 TA/AA genotype. In the multivariable analysis, the ESR, anti-CCP, and rs767649 TA/AA genotype remained significant predictors of RA susceptibility, with the TA/AA genotype showing a particularly strong association.

**Table 7:** Regression analysis of predictors of RA susceptibility p<0.05 is considered significant. OR: odds ratio; CI: confidence interval ESR: erythrocyte sedimentation rate; anti-CCP: anti-cyclic citrullinated peptide

	Univariable				Multivariable			
	p	OR	95% CI		P	OR	95% CI	
ESR	<0.001	1.214	1.132	1.302	0.001	1.148	1.062	1.241
Anti-CCP	<0.001	1.076	1.051	1.102	0.002	1.056	1.020	1.093
rs767649 TA+AA	0.004	0.492	0.302	0.801	0.038	2.537	1.932	6.909

The results of the regression analysis of the predictors of RA activity are displayed in Table 8. Univariable analysis indicated significant associations of RA activity with the ESR, anti-CCP, rs767649 TA/AA genotype, and disease duration. Multivariable analysis showed that the ESR, anti-CCP, and rs767649 TA/AA genotype significantly predicted RA activity, while age and disease duration did not.

**Table 8:** Regression analysis of predictors of RA activity p<0.05 is considered significant. RA: rheumatoid arthritis; B: regression coefficient; ESR: erythrocyte sedimentation rate; anti-CCP: anti-cyclic citrullinated peptide

	Univariable		Multivariable	
	B	P	B	p
Age	-0.016	0.339		
ESR	0.090	<0.001	0.048	<0.001
Anti-CCP	0.037	<0.001	0.011	<0.001
Duration	-0.269	<0.001	-0.003	0.943
rs767649 TA+AA	-1.312	<0.001	-0.142	0.002

## Discussion

This study sheds light on the potential association between miR-155 polymorphism and RA susceptibility and activity. Understanding the genetic factors that contribute to RA pathogenesis can aid in the development of effective diagnostic and therapeutic approaches, ultimately improving patient outcomes and quality of life.

The comparison of laboratory parameters indicated that the RA group exhibited significantly lower hemoglobin levels and higher ESR, serum creatinine, and anti-CCP levels than the control group. However, there were no significant differences in other parameters between the two groups. Analysis of clinical data of the RA group revealed a mean disease duration of eight years, with the majority of the patients showing moderate disease activity (63.6%). The presence of the rs767649 TA and AA genotypes was significantly lower in the RA group than in the control group.

In addition, the TA and AA genotypes showed a protective effect against the development of RA. In the RA group, the TT genotype was associated with higher anti-CCP levels and ESR than other genotypes. Furthermore, the clinical parameters, including disease duration, DAS, and readmission rate, varied significantly among the three genotypes. Furthermore, ESR, anti-CCP, and rs767649 TA/AA genotype exhibited significant associations with RA susceptibility both in the univariable and multivariable analyses. Regarding the prediction of RA activity, ESR, anti-CCP, and rs767649 TA/AA genotype were significantly associated with disease activity in both the univariable and multivariable analyses.

Overall, these findings indicated the potential link between miR-155 polymorphism and RA activity, highlighting the potential protective role of the TA and AA genotypes against the development of RA. Further research is needed to elucidate the mechanisms underlying this relationship. Our findings also emphasize the potential role of ESR, anti-CCP, and rs767649 TA/AA genotype as crucial predictors of both RA susceptibility and disease activity.

RA is a disease of unknown origin, characterized by many inflammatory changes affecting the joints, cartilage, bones, and, to some extent, extra-articular sites [14]. RA is a chronic inflammatory disorder in which miRNAs modulate the inflammatory process in the joints, with the potential to serve as biomarkers of both the inflammatory process and therapeutic response [15]. The intricate association observed between the rs767649 TA/AA genotype and an increased risk of RA in the multivariate analysis raises questions regarding the precise role of this genetic variant in RA pathogenesis. The miR-155 rs767649 polymorphism is known to influence key regulatory pathways within the immune system [16,17], potentially exacerbating the inflammatory responses characteristic of RA. This finding suggests a nuanced genetic landscape contributing to RA susceptibility, indicating the necessity for further investigations on the molecular mechanisms underlying the influence of this specific genotype on the complex interplay of immune dysregulations in RA.

In parallel, the significant correlations of elevated ESR and anti-CCP levels with RA susceptibility and disease activity underscore the pivotal roles of these biomarkers in shaping the disease trajectory. An elevated ESR level likely signifies the intensity of the systemic inflammatory process, serving as a reliable marker of disease activity and severity. Similarly, the high levels of anti-CCP, a specific biomarker of autoantibody-mediated immune responses, reflect the pathological progression and severity of RA, emphasizing its significance in early diagnosis and disease monitoring.

In this study, the median levels of serum creatinine and anti-CCP were statistically and significantly higher in the RA group than in the control group (P<0.001), in contrast with the findings of Li et al. (2022) who showed that serum creatinine levels were not significantly different between RA and control groups [18]. Li et al. (2022) also showed that additional laboratory and clinical variables, including RA-specific factors, DAS28-ESR clinical score, and various medical treatments, were not significantly different between the two groups [18].

Nowadays, various drug, physical, nonpharmacological, and surgical treatments are available for achieving remission and low disease activity [19]. Among these approaches, drug treatment, with disease-modifying anti-rheumatic drugs (DMARDs), nonsteroidal anti-inflammatory drugs, glucocorticoids, and biologics, is the most frequently used method [20]. However, RA cannot be cured using drugs. Owing to the insufficient response, adverse events, and exorbitant expense of the currently available therapeutic strategies, novel drugs for the treatment of RA are needed [21].

Clinical implications

Incorporating genetic screening for miR-155 polymorphism may help identify individuals at a high risk of developing RA, enabling early interventions and personalized treatment strategies. In addition, ESR and anti-CCP as predictors of RA activity could aid in the monitoring of disease progression and tailoring treatment plans accordingly.

Strengths and limitations

A notable strength of this study lies in its comprehensive analysis of both genetic and clinical parameters, providing a comprehensive understanding of the complex interplay between genetic predisposition and disease activity in RA. However, the study's limited sample size restricts the generalizability of the findings. In addition, the study was conducted on Egyptian patients only, which also limit the generalizability. Therefore, further multicentric prospective investigations with a larger sample size are required for the validation of these results.

## Conclusions

Our findings suggest that the rs767649 TA/AA genotype may confer a protective effect against RA development, while ESR and anti-CCP levels serve as valuable predictors of disease activity. MiR-155, especially the functional variant rs767649, plays a major role in susceptibility to the increased risk of RA, stressing the role of miR-155 as a therapeutic target in the treatment of Egyptian patients with RA. These insights highlight the potential for integrating genetic screening and monitoring of specific biomarkers in clinical practice, facilitating early detection and tailored management of RA. Further research is needed to validate these findings and elucidate the mechanisms underlying the observed associations.
